# FAR1/FHY3 Transcription Factors Positively Regulate the Salt and Temperature Stress Responses in *Eucalyptus grandis*

**DOI:** 10.3389/fpls.2022.883654

**Published:** 2022-05-04

**Authors:** Jiahao Dai, Jin Sun, Wenjing Peng, Wenhai Liao, Yuhan Zhou, Xue-Rong Zhou, Yuan Qin, Yan Cheng, Shijiang Cao

**Affiliations:** ^1^College of Forestry, Fujian Agriculture and Forestry University, Fuzhou, China; ^2^University Key Laboratory of Forest Stress Physiology, Ecology and Molecular Biology of Fujian Province, College of Forestry, Fujian Agriculture and Forestry University, Fuzhou, China; ^3^College of Agriculture, Fujian Agriculture and Forestry University, Fuzhou, China; ^4^Agriculture and Food, Commonwealth Scientific and Industrial Research Organisation (CSIRO), Canberra, ACT, Australia; ^5^Fujian Agriculture and Forestry University and University of Illinois at Urbana-Champaign School of Integrative Biology Joint Center for Genomics and Biotechnology, Fujian Provincial Key Laboratory of Haixia Applied Plant Systems Biology, College of Life Science, Ministry of Education, Fujian Agriculture and Forestry University, Fuzhou, China; ^6^Key Laboratory of Genetics, Breeding and Multiple Utilization of Corps, College of Life Science, Ministry of Education, Fujian Agriculture and Forestry University, Fuzhou, China; ^7^Guangxi Key Lab of Sugarcane Biology, College of Agriculture, Guangxi University, Nanning, China; ^8^State Key Laboratory for Conservation and Utilization of Subtropical Agro-Bioresources, College of Agriculture, Guangxi University, Nanning, China; ^9^College of Plant Protection, Fujian Agriculture and Forestry University, Fuzhou, China

**Keywords:** *FAR1/FHY3*, transcription factors, *Eucalyptus grandis*, salt stress, temperature stress

## Abstract

FAR-RED ELONGATED HYPOCOTYLS3 (FHY3) and its homolog FAR-RED IMPAIRED RESPONSE1 (FAR1), which play pivotal roles in plant growth and development, are essential for the photo-induced phyA nuclear accumulation and subsequent photoreaction. The FAR1/FHY3 family has been systematically characterized in some plants, but not in *Eucalyptus grandis*. In this study, genome-wide identification of *FAR1/FHY3* genes in *E. grandis* was performed using bioinformatic methods. The gene structures, chromosomal locations, the encoded protein characteristics, 3D models, phylogenetic relationships, and promoter *cis*-elements were analyzed with this gene family. A total of 33 *FAR1/FHY3* genes were identified in *E. grandis*, which were divided into three groups based on their phylogenetic relationships. A total of 21 pairs of duplicated repeats were identified by homology analysis. Gene expression analysis showed that most *FAR1/FHY3* genes were differentially expressed in a spatial-specific manner. Gene expression analysis also showed that *FAR1/FHY3* genes responded to salt and temperature stresses. These results and observation will enhance our understanding of the evolution and function of the *FAR1/FHY3* genes in *E. grandis* and facilitate further studies on the molecular mechanism of the *FAR1/FHY3* gene family in growth and development regulations, especially in response to salt and temperature.

## Introduction

The transposase-derived transcription factors FAR-RED ELONGATED HYPOCOTYL3 (FHY3) and its homolog FAR-RED-IMPAIRED RESPONSE1 (FAR1) are two best-characterized members from the FAR1-RELATED SEQUENCE (FRS) family, which control phyA signaling in a partially redundant manner (Wang and Deng, 2002). As a type of transcription factors derived from the ancient Mutator-like transposase, FAR1/FHY3s regulate the expression of the downstream genes by binding the FHY3/FAR1-binding site (FBS) *cis*-acting elements within their promoters in the form of homodimer or heterodimer (Lin and Wang, 2004). FAR1/FHY3s are considered as the founding members of the *FRS* and *FRS-RELATED FACTOR (FRF)* gene families, which are conserved among land plants (Ma and Li, 2018).

Light is one of the pivotal environmental signals that regulate plant growth and development. It has been reported that the loss-of-function mutants of *fhy1* together with *fhy2* and *fhy3* were shown in *Arabidopsis thaliana* (L.) to display an elongated hypocotyl in far-red (FR) light but not in white light (Whitelam et al., 1993), indicating their functions in light response in plants. Understanding the mechanisms by which the light controls the plant development has long been of great interest to plant biologists (Li et al., 2012). Plants can perceive and absorb the light of different quality, duration, and wavelength through different types of photoreceptors, including phytochromes (phys) (Chen et al., 2004; Li et al., 2010, 2011; Chen and Chory, 2011; Ma et al., 2016). In *Arabidopsis*, there are five *phys* designated *phyA* to *phyE*, encoding different phytochromes, which sense red (R) and FR light, and regulate various growth and development processes, including seed germination, low-quality growth, chlorophyll synthesis, stomatal membrane opening, and flower initiation (Shin et al., 2009; Franklin and Quail, 2010; Li et al., 2010; Wang and Wang, 2015). Phytochrome exists in two photo-interconvertible forms, i.e., a R-light-absorbing Pr form and a FR light-absorbing Pfr form. AtFHY3 and AtFAR1 are essential for the Pfr formation of phyB together with phyD and phyE to maintain a regular expression pattern of ELF4 after the sunset in short days (Siddiqui et al., 2016).

AtFHY3 and AtFAR1 form homodimers or heterodimers to directly bind promoters and activate the transcription of *AtFHY1* and *AtFHL*, which encode critical regulators of phyA translocating into the nucleus under FR light (Ma and Li, 2018). AtFHY3 and AtFAR1 are also involved in ultraviolet-B (UV-B) light signal transduction. In the presence of UV-B, they directly bind the promoter of *AtCOP1* and activate its transcription. A recent study in *Arabidopsis* reported that FHY3, rather than FAR1, functioned in early photomorphogenic UV-B response (Huang et al., 2012). Although UV-B (wavelength ranging from 280 to 315 nm) only represents a small part of sunlight, it exerts a strong influence on the growth and development of plants after reaching the surface of the Earth (Huang et al., 2012). Based on the studies on model plants, AtFHY3 and AtFAR1 are regarded as essential for phytochrome signaling. For example, the loss-of-function of FHY3 and FAR1 exhibited an exaggerated shade avoidance phenotype in *Arabidopsis* (Liu et al., 2019). It has also been proposed that these two proteins may have divergent roles likely through protein sub-functionalization (Lin et al., 2008). Loss-of-function of either AtFHY3 or AtFAR1 resulted in the loss-of-expression of the target genes (Hudson et al., 2003; Li et al., 2011).

AtFHY3 and AtFAR1 are playing crucial roles in responses to the dark–light transition in mature plants (Ma and Li, 2018). Loss-of-function of FHY3 and FAR1 resulted in an increased reactive oxygen species (ROS) accumulation and sensitivity to oxidative stress in various plant species (Letunic and Bork, 2018). Several previous studies have concluded that FHY3 and FAR1 are partially redundant in function, and FHY3 plays a more critical role than FAR1 in various plant species (Lin et al., 2007, 2008; Ouyang et al., 2011; Huang et al., 2012; Tang et al., 2012, 2013). The systematic biological analysis showed that the *FHY3/FAR1*-related gene family evolved into one or more dysfunctional MULE transposons in eukaryotic genomes (Feschotte and Pritham, 2007). Protein structure analysis revealed that FHY3 and FAR1 contain three main functional domains, including C2H2 zinc finger domain at N-terminal with DNA-binding ability, putative core transposase domain in middle-terminal regions, and SWIM zinc finger domain at C-terminal with transcriptional activation activity in *Arabidopsis* (Lin et al., 2007, 2008). FAR1 appears to act at the downstream of the regulation cascade as a link between the signal transduction pathway and the cell cycle in yeast (Chang and Herskowitz, 1990). Previous findings indicated that overexpression of FAR1 in *Arabidopsis* increased cell size and nuclear localization of *FHY3/FAR1* proteins (Hudson et al., 1999; Wang and Deng, 2002; Alberghina et al., 2004), inspiring the idea that AtFHY3/FAR1 might function as Mutator-like transposase-derived transcription factors (Lin et al., 2007). A recent genome-wide analysis suggested that FHY3 has numerous putative targets in *Arabidopsis*, implying that FHY3 might have broader functions in plant growth and development, most of which are unknown (Tang et al., 2013).

Eucalyptus (*Eucalyptus grandis* Hill ex Maiden) is a large evergreen tree species grown initially in Australia, Philippines, Papua New Guinea, Indonesia, and Timor. Some *E. grandis* trees were spread to other areas and then widely planted in short-rotation woodlots worldwide. Eucalyptus oil extracted from its leaves, fruit, buds, and bark has a wide range of antibacterial, antiseptic, antioxidant, anti-inflammatory along with anti-cancer effects. Therefore, it is traditionally used to treat respiratory diseases, common cold, influenza, and sinus congestion (Sebei et al., 2015; Sharifi-Rad et al., 2017). However, abiotic stresses, including salt, cold, and heat stresses, can generate numerous ROS in *E. grandis*, which is a major threat to the timber accumulation for *E. grandis*. Among 25 *CsFHY3/FAR1* genes in tea, there were 11 upregulated genes and 13 downregulated genes, 1 upregulated gene and 23 downregulated genes, and 9 upregulated genes and 10 downregulated genes under 200 mM NaCl, 4°C, and 40°C stresses, respectively (*p* < 0.05) (Liu et al., 2021). These suggested that *FHY3/FAR1* genes were responsive to abiotic stress in some species. Therefore, further functions on EgFAR1/FHY3 proteins are greatly necessary to be carried out.

At present, our understanding of FAR1/FHY3 protein family is mainly from *Arabidopsis*, in which 14 FAR1/FHY3 family members have been identified. In the present study, genome-wide identification of FAR1/FHY3 genes from *E. grandis* was performed using bioinformatic methods. We showed that these genes also responded to salt and temperature stresses, confirming FAR1/FHY3 had broader functions in addition to responding to light. It is envisaged that the findings in this study would not only improve our current understanding on the FAR1/FHY3 proteins in *E. grandis* and broad woody plant species as a whole but also provide a set of gene tools for their genetic improvement.

## Materials and Methods

### Gene Identification and Protein Characterization

The protein sequences of *E. grandis, Arabidopsis*, and poplar (*Populus trichocarpa* Torr & Gray) (Du et al., 2021) were downloaded from JGI database (https://genome.jgi.doe.gov). The structural domains of FAR1/FHY3s (PF03101, PF04434, and PF10551) were obtained from the Pfam database (http://www.ebi.ac.uk/Tools/hm). The sequences containing FAR1/FHY3 domain were screened by Hmmsearch 3.3.2 software with standard parameters (e-values < e−5). The candidates were further verified by conservative domain analysis with SMART (Letunic and Bork, 2018). Expasy (http://www.expasy.org) was used to analyze the physical and chemical properties of the FAR1/FHY3 proteins. In addition, WoLF PSORT (https://wolfpsort.hgc.jp) was used to predict the subcellular localization of the FAR1/FHY3 proteins.

### Conservative Motif, Structure, and 3D Modeling Analyses of FAR1/FHY3s

The assembly and annotation data of *E. grandis* (version 2.0) were downloaded from JGI database. Based on the genome annotation information, the structures of *FAR1/FHY3* genes in *E. grandis* were analyzed by GSDS (http://gsds.cbi.pku.edu.cn/) (Hu et al., 2015). MEME 5.4.1 software was used to analyze the conserved domain of FAR1/FHY3 proteins (Bailey et al., 2009). The maximum number of Motif discoveries was set to 15, whereas other parameters were the default values. The TBtools 0.9867 software was used for result visualization (Chen et al., 2020a). The three-dimensional (3D) structures of the *E. grandis* FAR1/FHY3 proteins were also determined using the AlphaFold 2.0 (Jumper et al., 2021). And their validations were assessed through Ramachandran plot analysis (Lovell et al., 2003). At the same time, the secondary structure characteristics of predicted proteins were resolved by DSSP2.1.0 (Kabsch and Sander, 1983).

### Evolutionary Analysis of FAR1/FHY3 Gene Family

The FAR1/FHY3 protein sequences of the above three species were aligned by Clustal Omega 1.2.4 software with default parameters (http://www.clustal.org/omega/) (Feschotte and Pritham, 2007). The phylogenetic assay was performed by IQ-TREE v2.0.3 for (maximum likelihood (ML) algorithm with the best-fit model “JTT + F + R5” and 1,000-bootstrap values.

### Chromosome Location and Homology Analysis of *FAR1/FHY3* Genes

Following the JGI database analysis, a chromosome localization map of the *FAR1/FHY3* genes was drawn by MapChart2.32 (Voorrips, 2002). Sequence homology among the members within the *FAR1/FHY3* family in *E. grandis* was analyzed by BLAST (https://blast.ncbi.nlm.nih.gov/Blast.cgi) and visualized by using Circos 0.69 software (http://circos.ca/) (Krzywinski et al., 2009).

### Promoter and Expression Analysis of *FAR1/FHY3* Genes

The 2,000 bp upstream sequences of *EgFAR1/FHY3s* were used to identify and sort their conserved *cis*-elements against the PlantCARE database (http://bioinformatics.psb.ugent.be/webtools/plantcare/html/) (Lescot et al., 2002). RNA-seq datasets were downloaded from the EucGenIE database (http://www.eucgenie.org/) (Hefer et al., 2011). The expression profiles of *FAR1/FHY3* genes in immature xylem, xylem, phloem, mature leaves, young leaves, and stem tips were calculated based on the read abundance. The results were visualized by pheatmap function in ComplexHeatmap 3.14 package (https://bioconductor.org/packages/release/bioc/html/ComplexHeatmap.html) (Gu et al., 2016) following data normalization.

### Plant Materials and Growth Conditions

*Eucalyptus grandis* clone (Eg5) seedlings were grown under natural conditions in the field laboratory, College of Forestry, Fujian Agriculture and Forestry University (119°14′E, 26°5′N). The relative humidity was about 77% and the light intensity, was 150 μmol m^−2^ s^−1^. The red soil for cultivation has organic matter content from 2.57% to 6.07% with the pH value 5.

### Expression Profile of *FAR1/FHY3* Genes Under Salt and Temperature Stress

Young *E. grandis* seedlings of 12 months old were treated with 100 and 200 mM NaCl for 0, 6, and 12 h for salt stress, with distilled water as the control. For the temperature stress test, *E. grandis* seedlings were placed under 4°C and 40°C, respectively, whereas the control group was kept at room temperature (25°C) for a period of 0, or 6 or 12 h. Tissue samples were collected immediately after the stress, and stored at −80°C for further analysis. For each biological replicate, six different seedlings were used, and the experiment was repeated at least three times. Total RNA and cDNA were obtained following the standard protocols as described in our previous study (Zhang et al., 2020). TransStart^®^Top Green qPCR SuperMix (Transgen, Beijing, China) and Bio-Rad CFX-96 detection system were used to conduct quantitative reverse transcriptase polymerase chain reaction (qRT-PCR) analysis. The β-actin was used as a reference gene (Sun et al., 2021). Relative transcript profile was calculated using the comparative 2^−ΔΔCT^ method (Livak and Schmittgen, 2001). All experiments were performed using three biological replicates and three technical replicates. The primers used in the study are listed in Supplementary Table S1.

## Results

### Identification of *FAR1/FHY3* Gene Family in *E. grandis*

Thirty-three *FAR1/FHY3* genes were identified in *E. grandis* and named as *EgFAR1*–*EgFAR33* following the order of appearance on chromosomes (Supplementary Table S2). All of them were located on chromosome-anchored scaffolds. The *FAR1/FHY3* genes were putatively encoding proteins of 211–880 amino acid residues with the minimum and maximum molecular weight of 24.08 and 99.65 kDa, respectively. The isoelectric points of EgFAR22 and EgFAR33 were the lowest (4.7094) and the highest (9.6928), respectively. Therefore, it was interesting to find the very large difference in protein sizes and molecular weights from *Arabidopsis*. The significant difference in isoelectric points of FAR1/FHY3 proteins suggested that they might be functioning at various acidity and basicity. To further investigate the functioning positions of the FAR1/FHY3 proteins, their subcellular localizations were predicted using WoLF PSORT. The results showed that 23 FAR1/FHY3 proteins were located in the nucleus, and 8 FAR1/FHY3 proteins were located in the cytoplasm or chloroplast. Furthermore, EgFAR11 and EgFAR28 were located in the cytoskeleton (Supplementary Table S2). The nucleus sub-localization of most FAR1/FHY3 proteins is consistent with the transcriptional activity of this gene family in plants.

### Phylogenetic Relationships of *FAR1/FHY3* Genes in *E. grandis*

**Figure 1:**
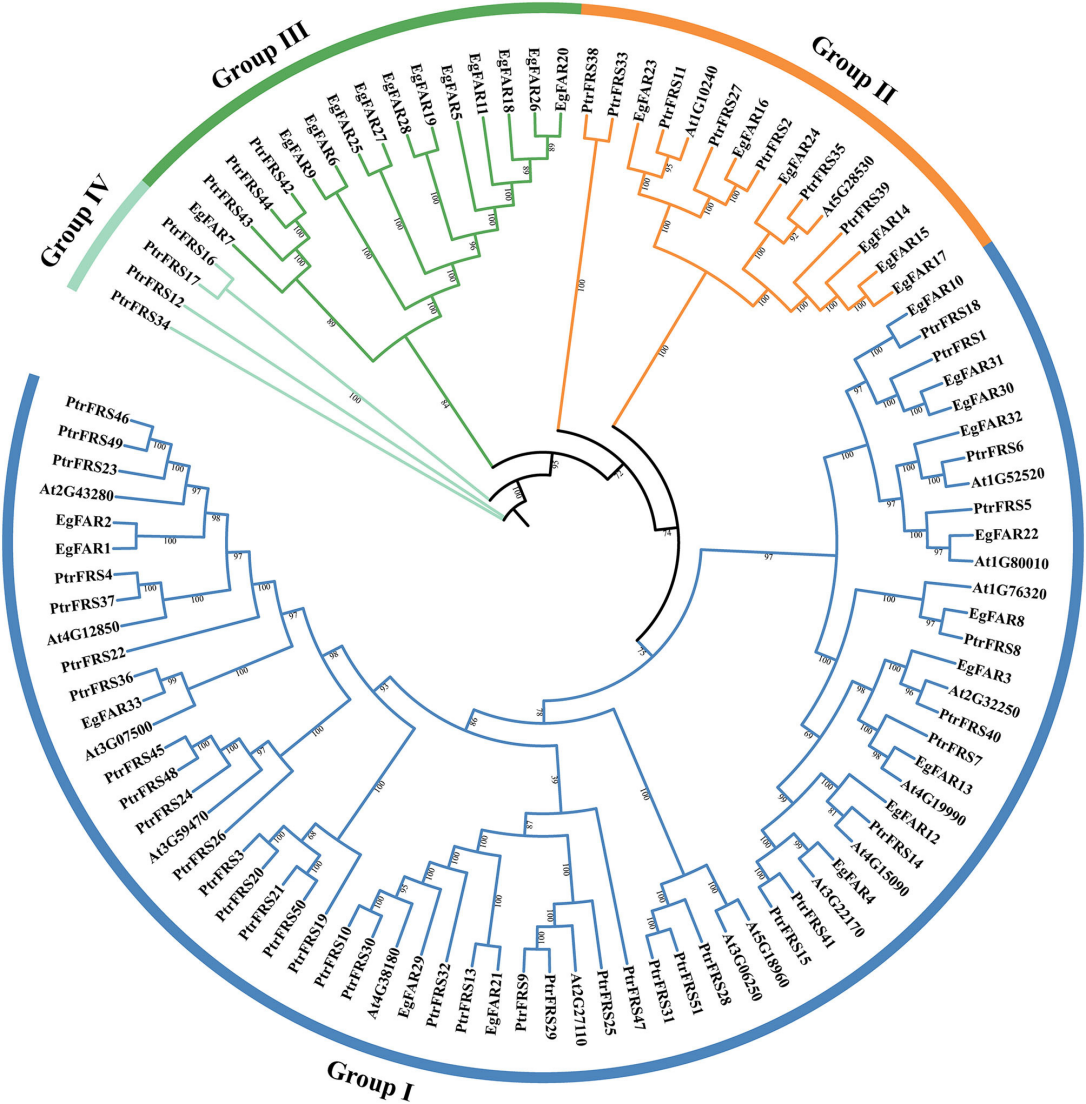
Phylogenetic tree of FAR1/FHY3 protein sequences in *Eucalyptus grandis, Arabidopsis*, and poplar. The prefix “Eg,” “At,” and “Ptr” stand for *E. grandis, Arabidopsis*, and poplar, respectively. “Group I,” “Group II,” “Group III,” and “Group IV” were symbolized by blue, orange, green, and cyan colored arcs, respectively.

The FAR1/FHY3 protein sequences of *E. grandis, Arabidopsis*, and poplar were aligned by Clustal Omega 1.24 software, and a phylogenetic tree was constructed using the ML method (Figure 1). According to the sequence similarity, FAR1/FHY3s from them could be divided into four groups. In Group I, there were 15, 15, and 37 FAR1/FHY3s from *E. grandis, Arabidopsis*, and poplar, respectively. The Group II included six, two, and seven FAR1/FHY3 proteins from *E. grandis, Arabidopsis*, and poplar, respectively. A total of 12 FAR1/FHY3 members from *E. grandis* were present in Group III, without any from *Arabidopsis*. However, Group IV did not hit in *E. grandis*. It was also found that Group I (15) and Group II (6) contained the most and the least FAR1/FHY3s, respectively. There was variation in protein molecular weight in all groups in both *Arabidopsis* and *E. grandis*.

### Chromosome Localization of *FAR1/FHY3* Genes

**Figure 2:**
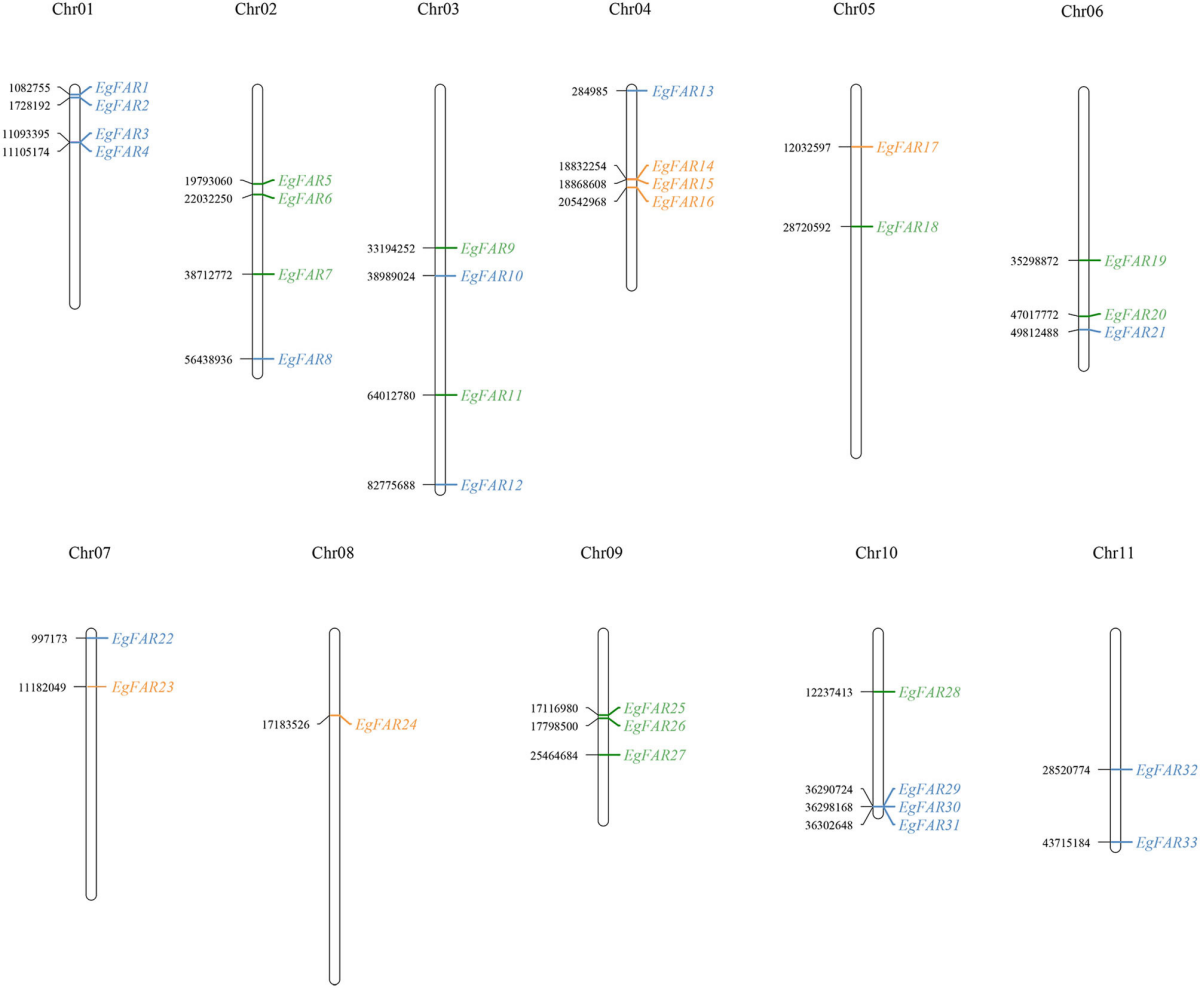
Chromosome locations of *FAR1/FHY3* genes on 11 *Eucalyptus grandis* chromosomes. The number of chromosomes was displayed at the top of each vertical line. The position data were shown on the left side of the chromosome and the gene name on the right side. Blue: *FAR1/FHY3* Group I; Orange: *FAR1/FHY3* Group II; Green: *FAR1/FHY3* Group III; chr: chromosome.

As shown in Figure 2, 33 genes were distributed on all the 11 chromosomes in *E. grandis* with 1–4 genes on each. From the perspective of chromosomal distribution, the chromosomes 1 and 11 only harbored Group I *FAR1/FHY3* genes, whereas chromosome 8 specifically carried Group II *FAR1/FHY3* genes. Half of *FAR1/FHY3* genes for Group II (3/6) were located on chromosome 4, whereas the genes for Group III were mainly (10/12) distributed on chromosomes 2, 3, 6, and 9. Taken together, the distribution of *FAR1/FHY3* genes on chromosomes is relatively even. However, the different groups tend to be localized on specific chromosomes.

### Gene Structure, Protein Motif Analyses of *FAR1/FHY3s*, and 3D Models of FAR1/FHY3 Proteins in *E. grandis*

**Figure 3:**
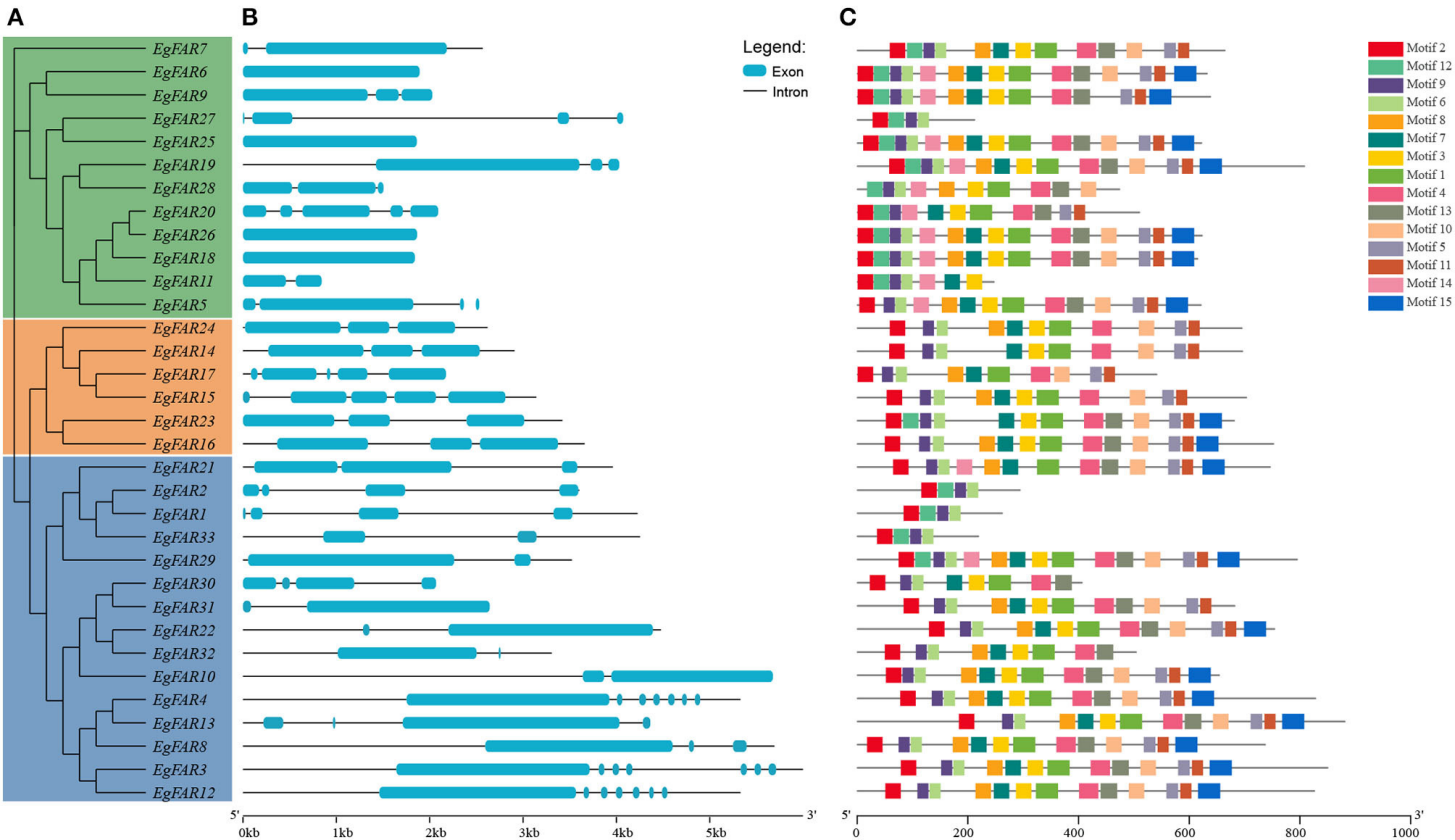
Conserved motifs of FAR1/FHY3 proteins and intron–exon organizations of *FAR1/FHY3* genes in *Eucalyptus grandis*. **(A)** The phylogenetic tree of *FAR1/FHY3* in *E. grandis*. **(B)** The intron–exon organizations of *FAR1/FHY3* genes. Blue boxes indicate exons; black lines indicate introns. The length of exons and introns for each *FAR1/FHY3* gene is proportionally displayed. **(C)** Conserved motifs of FAR1/FHY3 proteins. Motifs with specific colors can be found on the respective FAR1/FHY3 proteins. The order of the motifs corresponds to their position within individual protein sequences.

**Figure 4:**
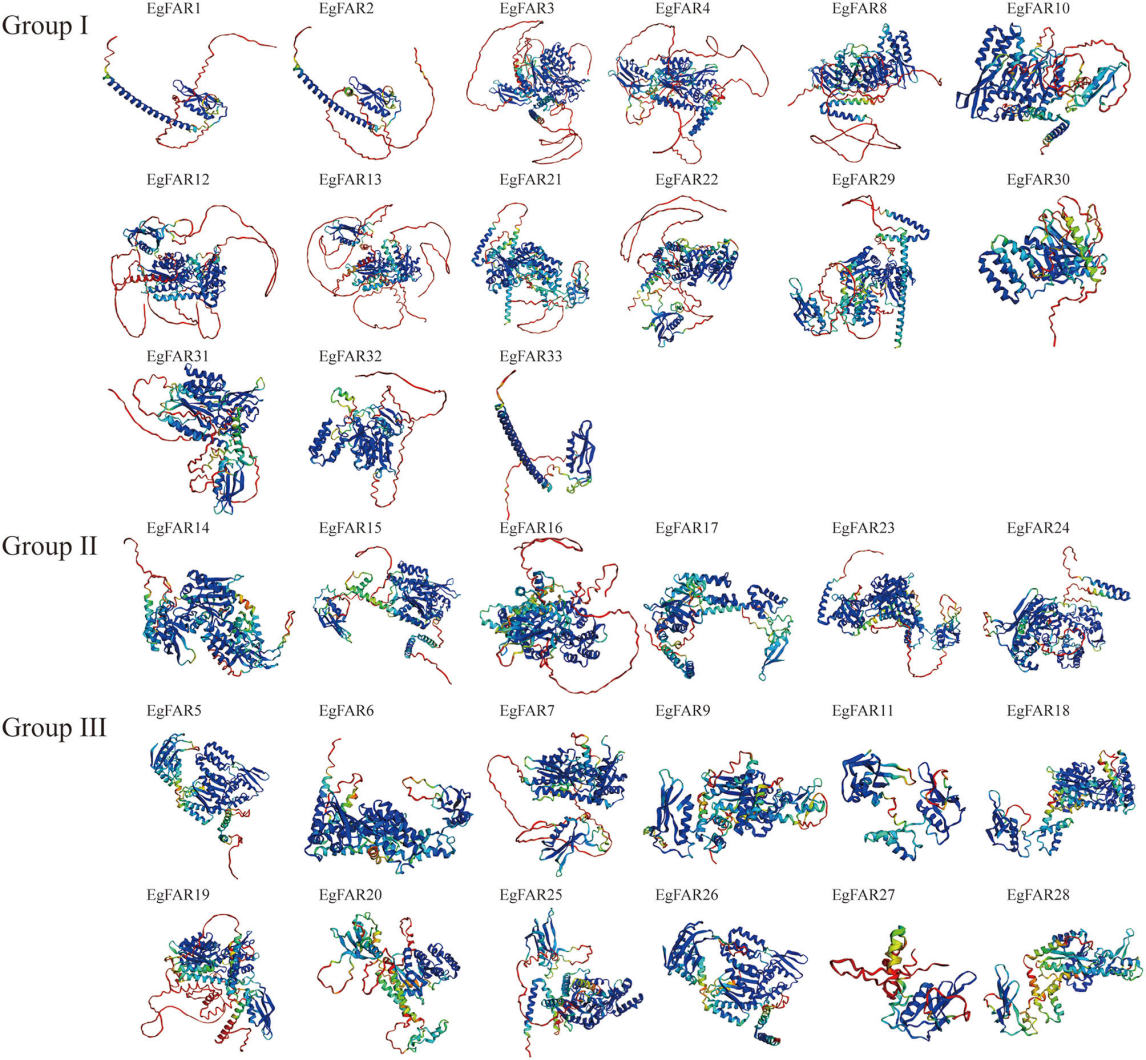
Three-dimensional models of EgFAR1/FHY3 proteins. Blue indicates high quality, whereas red indicates low quality.

The structure of proteins with conserved domains was analyzed by MEME (Figure 3). Gene structure analysis showed that the intron numbers of *FAR1/FHY3*s from different groups varied. Generally, there were four genes in Group III without introns, namely *EgFAR6, EgFAR18, EgFAR25*, and *EgFAR26*. The remaining genes contained one to eight introns. The Group II *FAR1/FHY3* genes had three to five introns, and the Group I *FAR1/FHY3* genes had relatively wide range of intron numbers, which were from one to eight (Figure 3B). As a result, 15 conserved motifs were found, namely Motifs 1–15. While most of the FAR1/FHY3 proteins harbored 15 common motifs with the order of 2_12_9_6_8_7_3_1_4_13_10_5_11_14_15, some FAR1/FHY3 proteins only harbored part of those motifs with specific motifs missing. For example, four FAR1/FHY3s (EgFAR1, EgFAR2, EgFAR27, and EgFAR33) only contained Motifs 2, 12, 9, and 6, whereas EgFAR7 lost specific motifs (Motif 14 and Motif 15). Generally speaking, for the motif feature of each group in *E. grandis*, Group III was more complex in structure, followed by Group I and Group II. Motif 9 occurred frequently in every protein, followed by Motif 2 (except for EgFAR28), and Motif 6 (except for EgFAR20) in *E. grandis* (Figure 3C; Supplementary Table S3). α-Helix and coil were the most frequently occurring structures. The Ramachandran plot analysis found that the characteristics of the EgFAR1/FHY3 protein models ranged from 84.6% to 99.8% in the model validation, implying that the predicted models were of excellent quality and dependability (Figure 4; Supplementary Table S4).

### Synteny Analysis of *FAR1/FHY3* Genes in *E. grandis*

**Figure 5:**
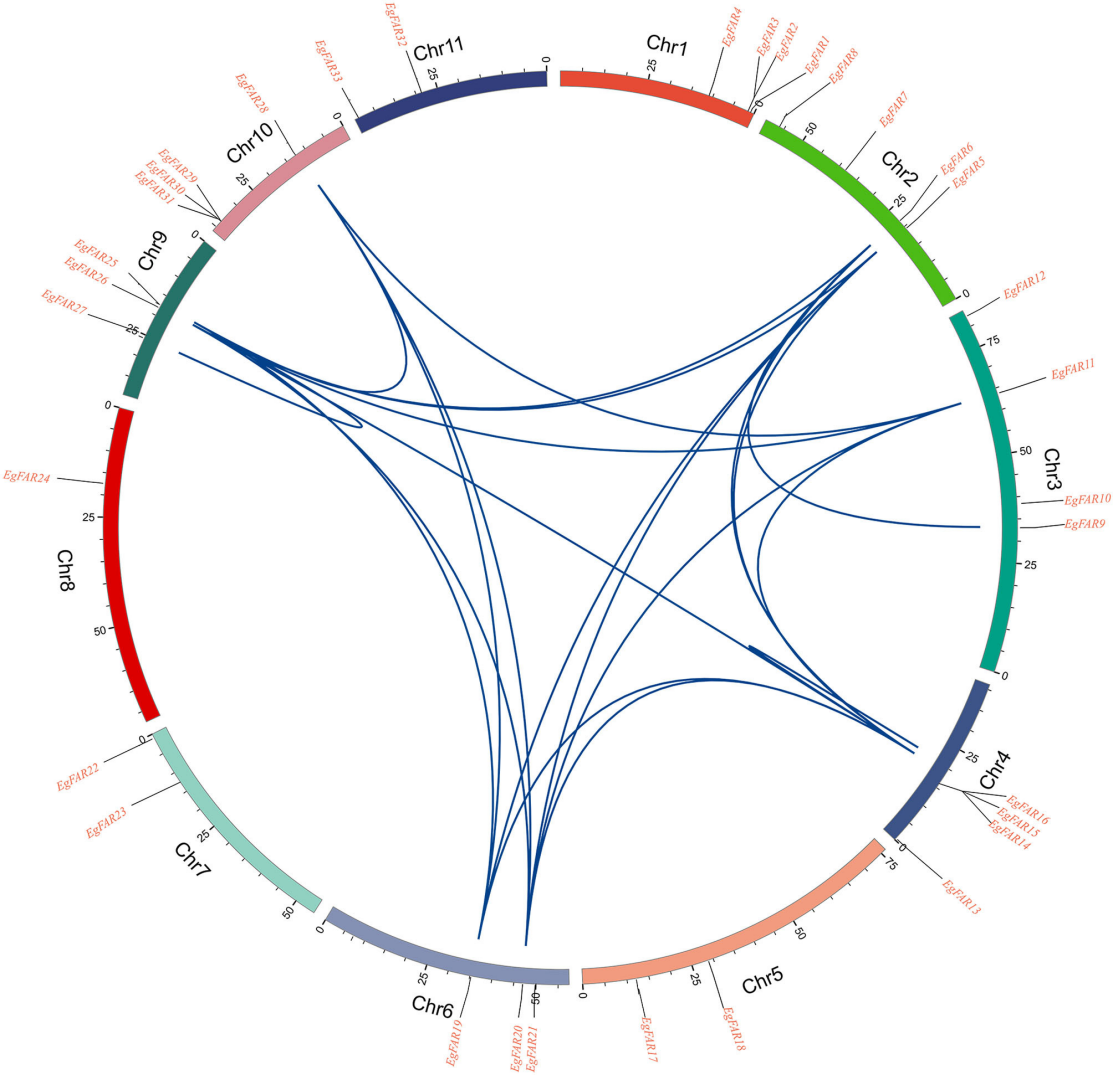
Homologous relationships of *FAR1/FHY3* family genes in eucalyptus. Small segments of different colors represent different chromosomes. The genes' positions on the chromosomes are shown in the corresponding positions on the scale above the small segments. The blue curve represents the correlation between the stages and the genes copied in tandem.

To investigate the evolution of the *FAR1/FHY3* genes in *E. grandis*, the segmental and tandemly duplicated gene pairs of *FAR1/FHY3* gene family was studied. Two tandem duplication pair (*EgFAR14*/*EgFAR16* and *EgFAR25/EgFAR27*) were identified on chromosomes 4 and 6 with a high similarity in coding sequence, which is located at close position on chromosome, respectively. On the other hand, 19 segmental duplication events were observed among the *FAR1/FHY3* genes. The segmental duplication gene pairs, such as *EgFAR5*/*EgFAR26, EgFAR6*/*EgFAR14, EgFAR14*/*EgFAR26*, and *EgFAR19*/*EgFAR14*, were located on separate chromosomes in the *E. grandis* genome (Figure 5; Supplementary Table S5). These results suggested that tandem and segmental duplication caused the expansion of the *FAR1/FHY3* gene family in *E. grandis*.

### The Promoter and Expression Analysis of *FAR1/FHY3* Family Genes in *E. grandis*

**Figure 6:**
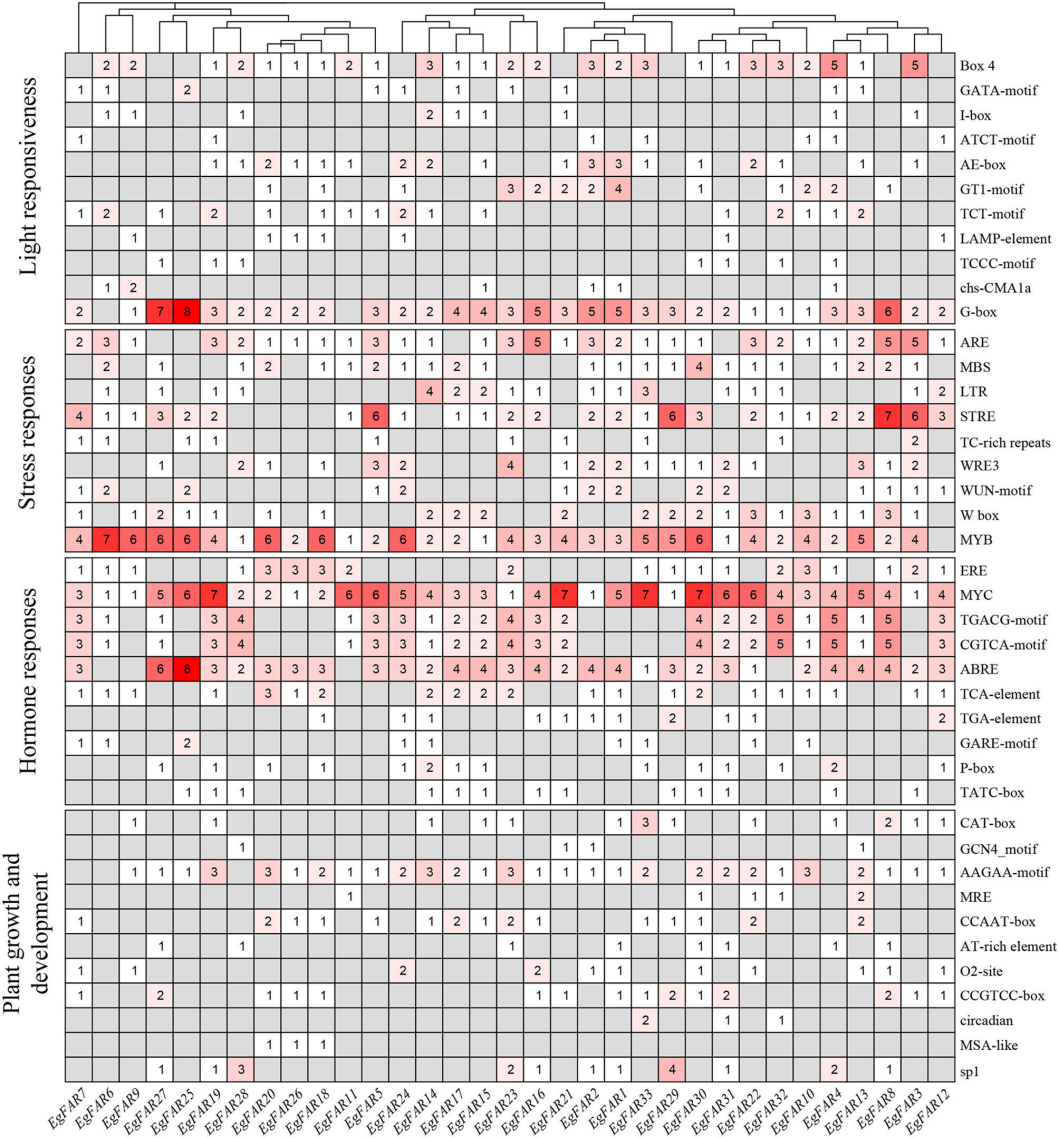
The numbers of *cis*-elements in the promoters of *EgFAR1/FHY3* genes.

In the present investigation (Figure 6), the promoters of *EgFAR1/FHY3* genes were seen to feature a diversity of *cis*-elements linked to phytohormones (10), abiotic stresses (9), light (11), and growth and development (11). MYC elements were observed in 33 *EgFAR1/FHY3* promoter regions, implying that they were the most prevalent. Other *cis*-elements identified in the promoter of *EgFAR1/FHY3* genes were MYB (32), G-box (31), ARE (29), and ABRE (29). The phytohormone type had the most *cis*-acting elements (463), including methyl jasmonate [MYC (136), TGACG-motif (61), CGTCA-motif (61)], abscisic acid [ABRE (93)], gibberellin [GARE-motif (10), P-box (16), TATC-box (13)], ethylene [ERE (31)], salicylic acid [TCA-element (29)], and auxin [TGA-element (13)]. The number of *cis*-elements (398) contained in the stress type was second only to phytohormone, such as high salt and low temperature [MYB (119)], low temperature [LTR (25)], defense and stress [TC-rich repeats (11), STRE (65)], and anaerobic induction [ARE (58)]. Moreover, a relatively large number of light-responsive *cis*-elements were observed, especially G-box and Box-4. Therefore, the appearance of them indicated that *EgFAR1/FHY3s* play a key role in stress response and especially in phytohormone, besides light responsiveness.

**Figure 7:**
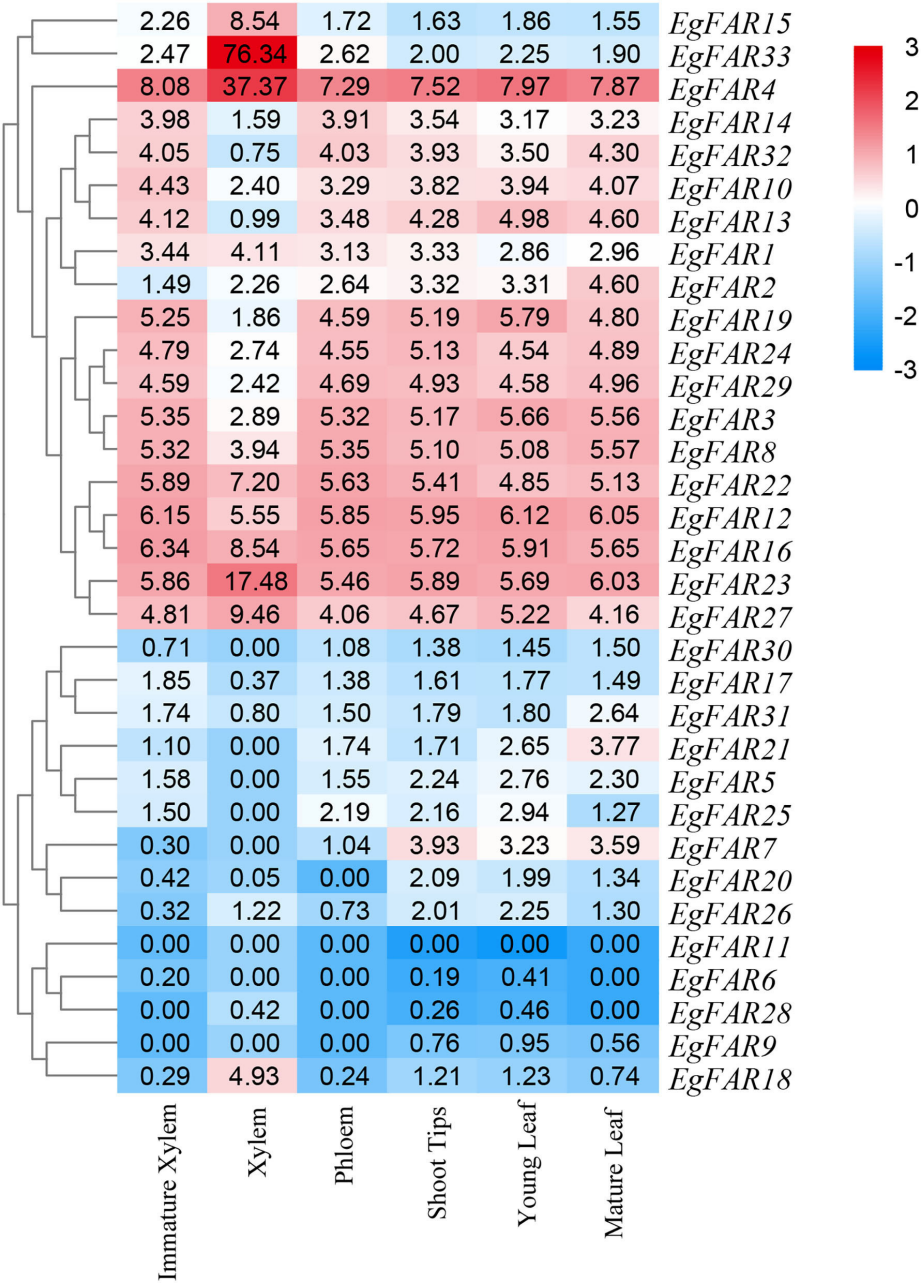
Tissue-specific expression profiles of *FAR1/FHY3* genes in *Eucalyptus grandis*. Expression profiles of *FAR1/FHY3* genes in immature xylem, xylem, phloem, shoot tips, young leaf, and mature leaf of *E. grandis* are displayed. Hierarchical clustering of expression profiles of *FAR1/FHY3* genes in different tissues and developmental stages. Red indicates high levels of transcript abundance, whereas blue indicates low levels. The color scale is shown on the right side.

Based on the expression data of *FAR1/FHY3* family genes in immature xylem, xylem, phloem, mature leaves, young leaves, and shoot tips, the expression profiles of *FAR1/FHY3* genes were obtained (Figure 7). The results showed that the gene members of specific *FAR1/FHY3* group had similar expression patterns in various parts of *E. grandis*. For example, most of the Group III members, such as *EgFAR22, EgFAR4*, and *EgFAR33*, were highly expressed in immature xylem and poorly expressed in mature leaves. Moreover, the majority of *FAR1/FHY3* genes in Group I, for example, *EgFAR11* and *EgFAR20*, exhibited relatively higher expression levels in phloem than those in other tissues. These observations indicated that the gene members in the same *FAR1/FHY3* group might have similar expression patterns and therefore similar functionalities in specific tissues.

In addition, *FAR1/FHY3* genes may be preferentially expressed in specific tissues. For example, in mature leaves, *EgFAR21* was highly expressed. In phloem, while the expression levels of *EgFAR10* and *EgFAR12* were low, the expressions of *EgFAR11* and *EgFAR20* were at relatively high levels. In shoot tip, *EgFAR7* and *EgFAR6* had high expression levels. Furthermore, in xylem, *EgFAR1* and *EgFAR31* had a high level of expressions. Among these *FAR/FHY3* genes, *EgFAR6* and *EgFAR7* were highly expressed in shoot tip, indicating that *EgFAR6* and *EgFAR7* might play an important role in the R-light regulation of *E. grandis*. In general, *FAR1/FHY3* family genes are expressed in various tissues at various developmental stages, playing critical roles in plant growth and development.

### The Expressions of Representative *FAR1/FHY3* Genes in Response to Salt and Temperature Stresses

**Figure 8:**
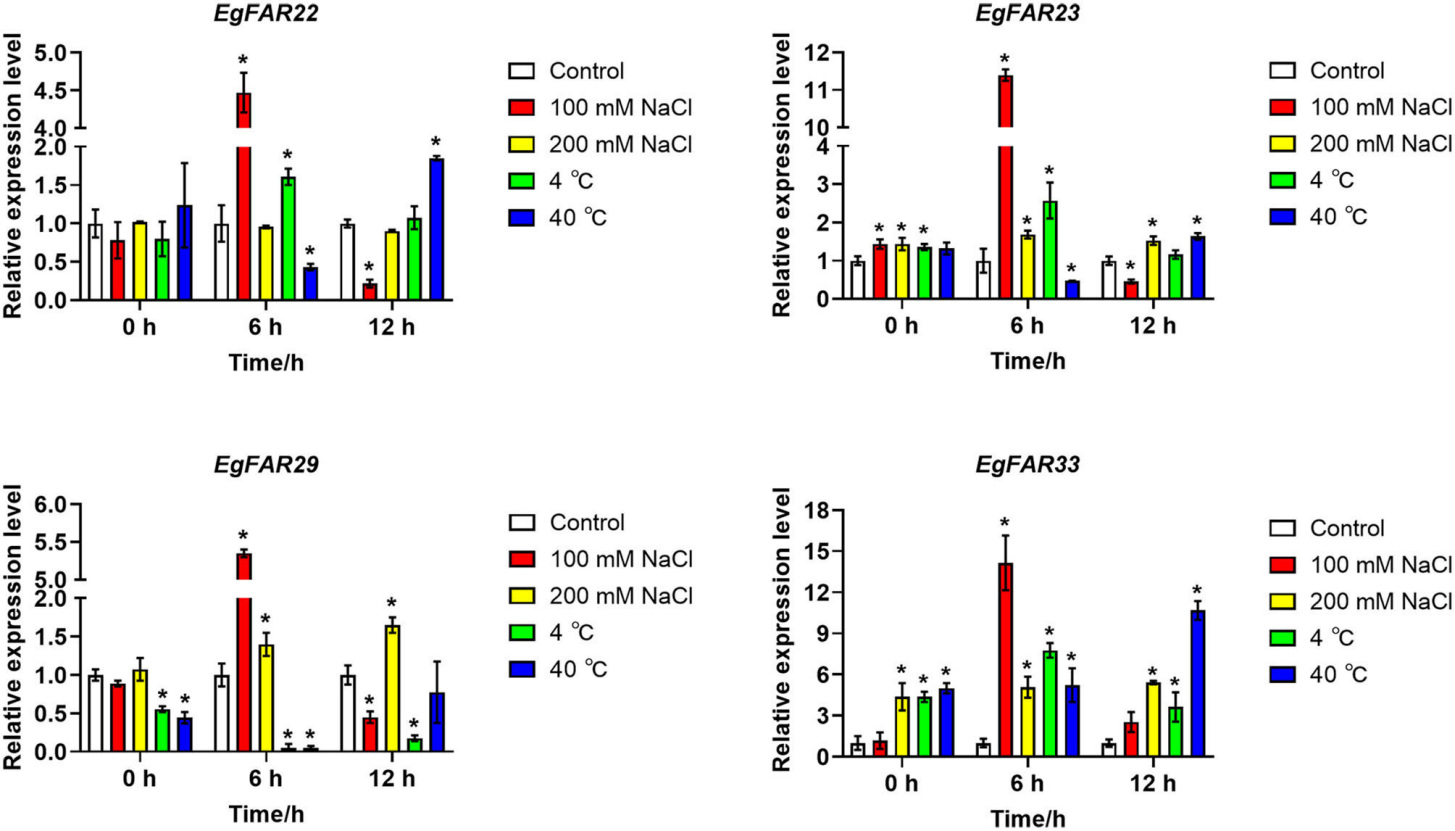
Expressions of four *FAR1/FHY3* genes in response to salt and temperature stresses. The qRT-PCRs were conducted after 0, 6, and 12 h of stress treatments. The low salinity was treated with 100 mM NaCl, and the high salinity was 200 mM NaCl, whereas the control was treated with distilled water. The low temperature was treated with 4°C and the high temperature was 40°C, whereas the control temperature was 25°C. Average data with standard errors is presented (*p < 0.05).

To investigate the responses of *FAR1/FHY3* gene family to other stresses such as salt and temperature stresses, four representative genes, *EgFAR22, EgFAR23, EgFAR29*, and *EgFAR33*, were selected for transcription observation. Among them, *EgFAR22, EgFAR23*, and *EgFAR29* were the highly expressed gene in young leaf, whereas *EgFAR33* was poorly expressed. Under 100 mM NaCl treatment, the expressions of all four selected *FAR1/FHY3* genes were significantly increased at 6 h but rapidly declined at 12 h. In contrast, the expressions of *EgFAR23, EgFAR29*, and *EgFAR33* were all upregulated at 12 h treatment by 200 mM NaCl. These results indicated that *FAR1/FHY3* genes were early-responsive genes under salt stress. Moreover, the response of those genes was much more sensitive under 100 mM than 200 mM NaCl treatment. Under cold treatment, the expressions of *EgFAR33* were significantly upregulated, whereas those of *EgFAR29* were downregulated at 6 and 12 h. The expression of *EgFAR23* was considerably higher than the control at 6 h, but this expression difference was diminished at 12 h. Under heat treatment, although the expression of *EgFAR33* showed a continuous increase tendency from 6 to 12 h, the expressions of the other three *FAR1/FHY3* genes decreased at 6 h and increased at 12 h. Moreover, after 12 h of heat shock, the expressions of four genes investigated (expecting *EgFAR29*) were significantly higher than the control, suggesting the involvement of this gene family in response to heat shock (Figure 8; Supplementary Table S6).

## Discussion

*Eucalyptus grandis* is a fast-growing evergreen tree belonging to the Myrtaceae family with a wide-spreading and relatively thin crown. Usually *E. grandis* grows 40–55 m high but exceptionally to 75 m (Myers et al., 1996). *Eucalyptus grandis* is one of the most important commercial trees widely used as the sources of wood, paper materials, and forage. Due to its characteristics of fast-growing and various usages, *E. grandis* has become a global renewable resource of fiber and energy. *Eucalyptus grandis* also has strong adaptability, especially to salt and drought stresses, making it an ideal ecological plant species being planted in a wide range of areas (Young, 1983; Lahr et al., 2018; Chen et al., 2020b). Moreover, its metabolites, for example, terpenes acting as defensive substance, are now used as unique pharmaceutical oils. It has been demonstrated that *E. grandis* has the highest genes diversity for those specialized metabolites (Ma and Li, 2018). In 2014, the genome of *E. grandis* was sequenced and released (Myburg et al., 2014), which facilitated and accelerated the functional and comparative genomic studies.

Genetic studies have identified two pairs of homologous genes essential for phyA signaling: *FAR1/HY3* and *FHY1/HL*, which mediate the light-dependent nuclear accumulation of phyA (Lin et al., 2007). In model plants *Arabidopsis* and rice, *FHY3/FAR1* has been confirmed to be involved in phytochrome signaling by controlling the phytochrome accumulation through FHY1 (Genoud et al., 2008; Lao et al., 2011). However, the specific biochemical function of FHY3 and FAR1 is still ambiguous and needs to be elucidated (Lin et al., 2007). In this study, genome-wide identification of *FAR1/FHY3* gene family was conducted in *E. grandis* using bioinformatic methods.

Comparing with the protein sizes and molecular weights in *Arabidopsis*, their large difference in *E. grandis* (Supplementary Table S2) might suggest new functions different from their homologies in *Arabidopsis*. Similar to *R2R3-MYB* (Soler et al., 2015), *NAC* (Hussey et al., 2015), *lignin* (Carocha et al., 2015), and *FLA* (MacMillan et al., 2015) gene families, it was interesting to observe more *FAR1/FHY3* members (33) than *Arabidopsis* (Figure 1) and 21 pairs of duplicated repeats (Figure 5), which is common to find duplicated genes in *E. grandis*. Moreover, as a Mutator-like transposase-derived transcription factor, maybe it will accelerate this process partly (Hudson et al., 2003; Feschotte and Pritham, 2007; Chae et al., 2021).

It seems that the segmental duplication (Figure 5) has been extended to the members of *FAR1/FHY3* genes in *E. grandis*, and mutations in gene structure (Figure 3) may affect the function of new members (Abdullah et al., 2021; Musavizadeh et al., 2021). Genome-wide identification of *FHY3/FAR1* family in other plants has rarely been reported, and the function of *FHY3/FAR1* genes in other biological processes other than phytochrome signaling remains to be elucidated (Lin et al., 2007). Recently, several studies indicated multifaceted roles of *FHY3/FAR1* in diverse physiological and developmental processes, such as plant architecture and flowering, chloroplast biogenesis and chlorophyll biosynthesis, circadian clock entrainment, UV-B signaling, ABA signaling, ROS homeostasis, and programmed cell death (PCD) (Lin and Wang, 2004; Allen et al., 2006; Li et al., 2011; Ouyang et al., 2011; Huang et al., 2012; Stirnberg et al., 2012; Gao et al., 2013; Tang et al., 2013; Kiseleva et al., 2017).

**Figure 9:**
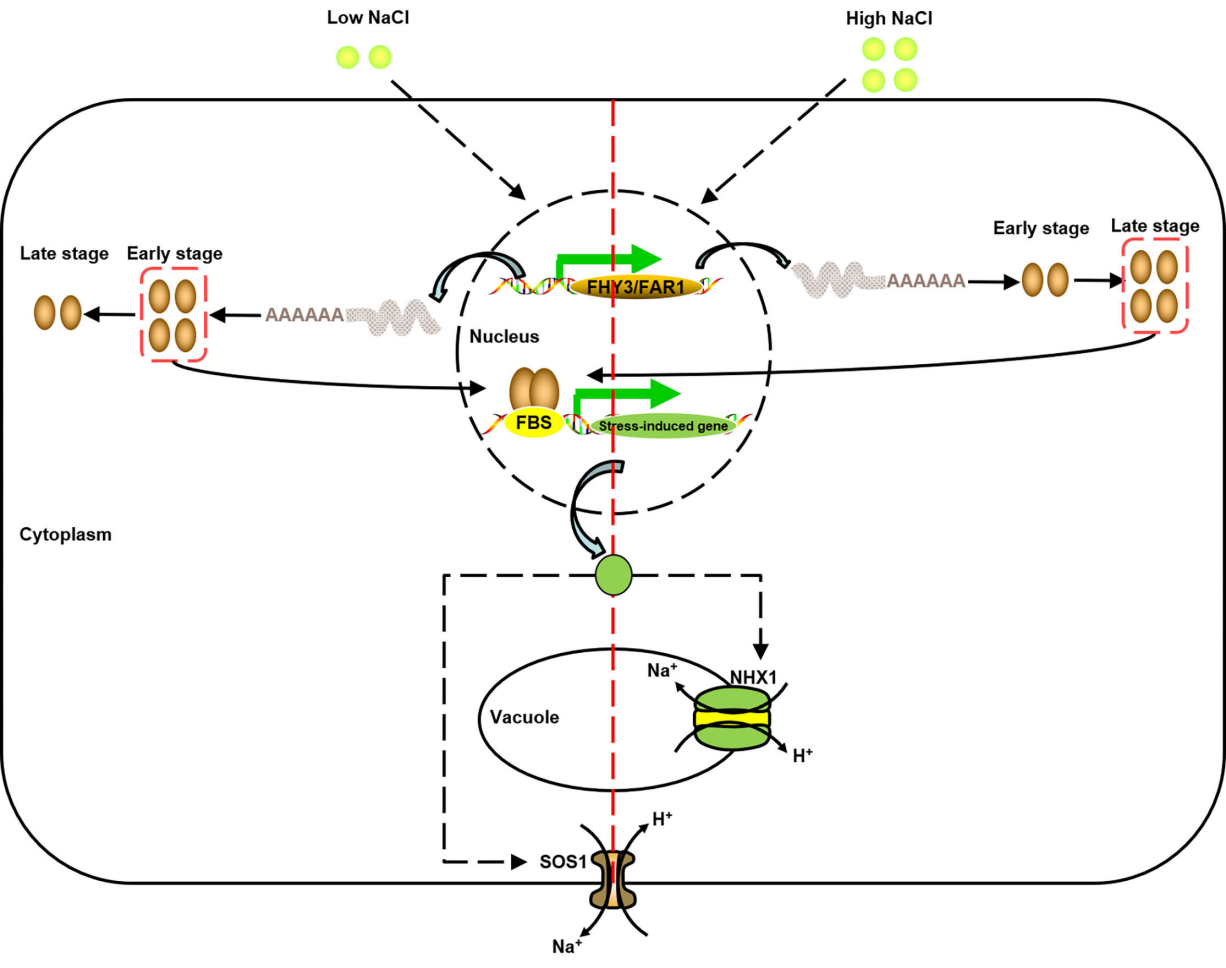
A model of the function of *FAR1/FHY3* under salt stress (modified from Halfter et al., 2000; Shi et al., 2002; Zhou et al., 2018). Under high/low salt stress, due to different response activities, different *FAR1/FHY3* genes were activated successively to further activate downstream salt stress-induced genes, and then achieved Na^+^ balance inside and outside the vacuole and cell membrane through SOS pathway.

Studies in *Arabidopsis* and rice suggested that *FHY3/FAR1*s control phytochrome accumulation through regulating the expression of *FHY1* (Lao et al., 2011). G-box and Box-4 indicated that the *FAR1/FHY3* genes in *E. grandis* might involve in the photoreceptive response (Figure 6). There was a link between photoresponse and response to salt/temperature stress, for example, the *EgFAR33* promoter had 3 Box-4s, 3 LTRs, and 5 MYBs (Figure 6) and more importantly, it was upregulated to salt/temperature stress (Figure 8). However, this link was ambiguous and needs more research. Fixed growth of plants forces them to evolve many adapting mechanisms to environmental stimuli, among which abiotic stresses are challenging plants all the time. In the presence of stresses, plants adjust their growth and development for surviving through various pathways, such as cell cycle regulation and hormone regulation. Additionally, the regulation of photosynthesis is another strategy for plants to respond to stresses. Publicly available microarray data have shown a significant expression of *FHY3/FAR1* in response to biotic/abiotic stresses, indicating some potential functions of *FAR1/FHY3* genes in plants (Wang and Deng, 2002; Allen et al., 2006; Stirnberg et al., 2012). High soil salinity is considered as a major abiotic threat for agricultural productivity in semi-arid or coastal areas (Espinosa-Ruiz et al., 1999). Previous studies showed that more than half of *C3H, WRKY*, and *FAR1* family genes were upregulated in response to salt stress, suggesting that these transcription factor families may participate in salt response and tolerance (Mansouri et al., 2019). In this study, we investigated the expression profiles of representative *FAR1/FHY3* genes after salt treatments and showed that *FAR1/FHY3s* were a group of the early-responsive genes in *E. grandis* under salt treatment. Interestingly, under 200 mM of salt concentration, the relative expression level of the *FAR1/FHY3* gene started to upregulate following the treatment for 12 h (Figure 8), indicating that *FAR1/FHY3* genes show a lagged response when plants are treated with a relative stronger salt stress (Figure 9).

Temperature is another critical environmental factor regulating plant growth and development (Penfield, 2008). In the current study, the expression of the most *FAR1/FHY3* genes did not change significantly in response to the cold treatment. However, under 40°C heat stress, the expressions of four *FAR1/FHY3* genes were significantly increased (Figure 8). This suggested that the *FAR1/FHY3* genes might have been involved in the responses to heat shock rather than the cold stress, despite of their lagged response under high-temperature stress. In conclusion, *FAR1/FHY3* genes in *E. grandis* showed a diverse expression in response to abiotic stresses, indicating that they are involved in different cellular processes and cell signaling (Faraji et al., 2021; Heidari et al., 2021).

## Conclusion

In *E. grandis*, abiotic stresses, especially salt and temperature stresses, are increasingly affecting wood productivity. Using bioinformatics methods and expression profile analysis, we identified *FAR1/FHY3* genes and showed they were responsive to salt treatment. In addition, *EgFAR33* showed great potential for understanding on the response to temperature stress. This study could provide potential to wood yield increase.

## Data Availability Statement

The datasets presented in this study can be found in online repositories. The names of the repository/repositories and accession number(s) can be found in the article/Supplementary Material.

## Author Contributions

YC and SC conceived this experimental study. JD performed most of the experiments and wrote the manuscript. JS was involved in all the bioinformatics and their visual analysis. WP, WL, and YZ analyzed some of the data. X-RZ made key comments on the revision of the manuscript. X-RZ, YQ, YC, and SC were involved in revising and improving the manuscript. All authors contributed to the article and approved the final version of the manuscript.

## Funding

This work was supported by National Natural Science Foundation of China (32170380), Fujian Agriculture and Forestry University Forestry Peak Discipline Construction Project (71201800739), and the Science and Technology Program of Fujian Province (2019N5008).

## Conflict of Interest

The authors declare that the research was conducted in the absence of any commercial or financial relationships that could be construed as a potential conflict of interest.

## Publisher's Note

All claims expressed in this article are solely those of the authors and do not necessarily represent those of their affiliated organizations, or those of the publisher, the editors and the reviewers. Any product that may be evaluated in this article, or claim that may be made by its manufacturer, is not guaranteed or endorsed by the publisher.
