# Supplementary material for: FAR1/FHY3 Transcription Factors Positively Regulate the Salt and Temperature Stress Responses in Eucalyptus grandis
Source: Front Plant Sci. 2022 May 4;13:883654. doi: 10.3389/fpls.2022.883654 (PMC9115564; doi:10.3389/fpls.2022.883654)
Supplement: Supplementary file 1 [file Table_1.DOCX]

**Supplementary Table 1.** The primers used in RT-qPCR

| Primer name | Primer sequence |
| --- | --- |
| Q-*EgFAR22*-F | CGCTTGTATGTACCGGAATTTG |
| Q-*EgFAR22*-R | CCTTCCTCCACAACCCTTATG |
| Q-*EgFAR23*-F | AGTCCAAGGAGAGGCTAGAA |
| Q-*EgFAR23*-R | GTCTCTTAGAACCAGCGAAGAC |
| Q-*EgFAR29*-F | CTCTCAAATCCAAGCTGCAATC |
| Q-*EgFAR29*-R | GAGCTTCATTTGTGACCTTTCC |
| Q-*EgFAR33*-F | GCGTCGGCTTGTCTGTAATA |
| Q-*EgFAR33*-R | ATTGCCTTACAGCCTTCTCTC |
| Q-*EgACTIN*-F | TTACCGCGATCTGGATCTTTAC |
| Q-*EgACTIN*-R | CCTGCATAAGCCTCCTTCAA |
