# Supplementary material for: FAR1/FHY3 Transcription Factors Positively Regulate the Salt and Temperature Stress Responses in Eucalyptus grandis
Source: Front Plant Sci. 2022 May 4;13:883654. doi: 10.3389/fpls.2022.883654 (PMC9115564; doi:10.3389/fpls.2022.883654)
Supplement: Supplementary file 2 [file Table_2.DOCX]

**Supplementary Table 2.** The characteristics of 33 *FAR1/FHY3* genes in *E. grandis*

| Gene name | Gene ID | Chromosome location | | | No. of amino acid | Protein molecular weight (MW; Da) | Protein isoelectric point (PI) | Predicted Subcellular location |
| --- | --- | --- | --- | --- | --- | --- | --- | --- |
| *EgFAR1* | Eucgr.A00281 | Chr01 | 1082755 | 1086981 | 261 | 29393.7 | 9.4365 | Chloroplast |
| *EgFAR2* | Eucgr.A00087 | Chr01 | 1728192 | 1731795 | 293 | 33250 | 9.2699 | Chloroplast |
| *EgFAR3* | Eucgr.A00910 | Chr01 | 11093395 | 11099392 | 827 | 94993.7 | 7.8444 | Nuclear |
| *EgFAR4* | Eucgr.A00908 | Chr01 | 11105174 | 11110502 | 849 | 97155.5 | 7.1653 | Nuclear |
| *EgFAR5* | Eucgr.B01259 | Chr02 | 19793061 | 19795591 | 620 | 71339.2 | 8.5342 | Nuclear |
| *EgFAR6* | Eucgr.B01589 | Chr02 | 22032250 | 22034142 | 631 | 72653.4 | 7.933 | Nuclear |
| *EgFAR7* | Eucgr.B01750 | Chr02 | 38712773 | 38715340 | 663 | 75443.3 | 8.334 | Nuclear |
| *EgFAR8* | Eucgr.B03670 | Chr02 | 56438941 | 56444634 | 736 | 85189.5 | 6.8148 | Nuclear |
| *EgFAR9* | Eucgr.C01967 | Chr03 | 33194251 | 33196279 | 637 | 73115.4 | 8.8039 | Cytoplasm |
| *EgFAR10* | Eucgr.C02098 | Chr03 | 38989024 | 38994700 | 653 | 76079.6 | 6.8111 | Nuclear |
| *EgFAR11* | Eucgr.C03166 | Chr03 | 64012779 | 64013621 | 246 | 28360.6 | 7.6692 | Cytoskeleton |
| *EgFAR12* | Eucgr.C04090 | Chr03 | 82775685 | 82781014 | 825 | 94820.8 | 7.1434 | Nuclear |
| *EgFAR13* | Eucgr.D00033 | Chr04 | 284985 | 289348 | 880 | 99649.7 | 8.9952 | Nuclear |
| *EgFAR14* | Eucgr.D01451 | Chr04 | 18832254 | 18835162 | 695 | 79835.9 | 6.6291 | Nuclear |
| *EgFAR15* | Eucgr.L00168 | Chr04 | 18868607 | 18870781 | 702 | 80525.7 | 7.8394 | Nuclear |
| *EgFAR16* | Eucgr.K03522 | Chr04 | 20542969 | 20546111 | 751 | 85439.6 | 7.351 | Chloroplast |
| *EgFAR17* | Eucgr.D01455 | Chr05 | 12032597 | 12034438 | 540 | 62647 | 6.4118 | Nuclear |
| *EgFAR18* | Eucgr.E01219 | Chr05 | 28720591 | 28720938 | 614 | 71287.9 | 8.1441 | Cytoplasm |
| *EgFAR19* | Eucgr.B00042 | Chr06 | 35298870 | 35302900 | 807 | 93751.9 | 7.869 | Nuclear |
| *EgFAR20* | Eucgr.F00557 | Chr06 | 47017772 | 47021734 | 509 | 58678.8 | 9.1099 | Nuclear |
| *EgFAR21* | Eucgr.F03610 | Chr06 | 49812490 | 49816966 | 745 | 83831.2 | 8.1659 | Nuclear |
| *EgFAR22* | Eucgr.F03904 | Chr07 | 997173 | 1000594 | 753 | 86568.3 | 4.7094 | Nuclear |
| *EgFAR23* | Eucgr.G00085 | Chr07 | 11182049 | 11182366 | 680 | 78220 | 7.7173 | Cytoplasm |
| *EgFAR24* | Eucgr.H00883 | Chr08 | 17183526 | 17185388 | 694 | 80014 | 7.1152 | Nuclear |
| *EgFAR25* | Eucgr.H01407 | Chr09 | 17116981 | 17118846 | 621 | 72153.4 | 7.6246 | Nuclear |
| *EgFAR26* | Eucgr.I00824 | Chr09 | 17798501 | 17802573 | 622 | 72246.9 | 8.3022 | Cytoplasm |
| *EgFAR27* | Eucgr.I00868 | Chr09 | 25464684 | 25468207 | 211 | 24081.5 | 6.5053 | Nuclear |
| *EgFAR28* | Eucgr.J01116 | Chr10 | 12237413 | 12238918 | 473 | 55334.2 | 7.9566 | Cytoskeleton |
| *EgFAR29* | Eucgr.I01547 | Chr10 | 36290723 | 36292791 | 794 | 90639.3 | 6.514 | Nuclear |
| *EgFAR30* | Eucgr.J03074 | Chr10 | 36298166 | 36300808 | 405 | 46582 | 6.5694 | Chloroplast |
| *EgFAR31* | Eucgr.J03076 | Chr10 | 36302646 | 36305953 | 681 | 78408 | 6.3878 | Nuclear |
| *EgFAR32* | Eucgr.J03078 | Chr11 | 28520776 | 28525028 | 503 | 57109.8 | 8.8677 | Nuclear |
| *EgFAR33* | Eucgr.K02085 | Chr11 | 43715186 | 43718846 | 218 | 24923.4 | 9.6928 | Nuclear |
